# Hospital Outcomes in Uninsured Patients With Disease and Disorders of Nervous System: A National Cohort Study During a Decade in the United States

**DOI:** 10.7759/cureus.13702

**Published:** 2021-03-04

**Authors:** Ali Seifi, Maryam Bahadori, Zahra Gheibi, Skyler L Kanegi, Alireza Mirahmadizadeh

**Affiliations:** 1 Department of Neurosurgery, University of Texas Health Science Center at San Antonio, San Antonio, USA; 2 Department of Neurology, Glenn Biggs Institute for Alzheimer's and Neurodegenerative Diseases, University of Texas Health Science Center San Antonio, San Antonio, USA; 3 Department of Epidemiology, Shiraz University of Medical Sciences, Shiraz, IRN; 4 Department of Medicine, University of Texas Health Science Center at San Antonio, San Antonio, USA; 5 Department of Epidemiology and Public Health, Non-Communicable Diseases Research Center, Shiraz University of Medical Sciences, Shiraz, IRN

**Keywords:** health insurance, insurance coverage, nervous system diseases, mortality, uninsured patients, healthcare insurance, medicare data

## Abstract

Objectives

Health insurance is associated with better outcomes in the admitted patient population, even after adjusting for other factors such as race and socioeconomic status. However, the literature is limited on the relationship between insurance status and hospital outcomes in patients hospitalized with the disease of nervous system.

Methods

This cross-sectional study used the Nationwide Inpatient Sample (NIS) database to achieve the results. All Major Diagnostic Category (MDC) codes from patients discharged for disease and disorders of nervous system between the years 2005 to 2014 were queried and analyzed for the impact of lack of insurance on patient outcome.

Results

Among 4,737,999 discharges, 5.6% had no insurance. The hospital mortality rate among uninsured and insured patients was 4.1% and 3.7%, respectively (P<0.001). In the multivariate analysis, hospital mortality of uninsured patients was higher in the elderly (aOR: 4.74[CI:4.52-4.97], P<0.001), those with comorbidities (aOR: 2.23[CI:2.18-2.27], P<0.001), Asians (aOR: 1.16[CI:1.12-1.20]. P<0.001), in rural areas (aOR: 1.44[ 95%CI:1.41-1.48], P<0.001) and those in the lowest household income quartile (aOR: 1.03[CI:1.01-1.05], P<0.001). The average length of stay (LOS) was shorter for the uninsured (4.79±8.26 vs 4.96±7.55 days, P<0.001).

Conclusions

The findings suggest that lack of health insurance is correlated with hospital mortality in patients hospitalized with disease and disorders of nervous system, with an increased disparity in vulnerable populations.

## Introduction

According to a 2019 Gallup poll, 25% of Americans delayed treatment for a serious condition due to costs, compared to 12% of Americans in 2001. The trend persists across all income groups with the greatest increase in the $40,000-$100,000 income range [1]. As legislators continue to discuss health insurance policy changes, research is needed to quantify the health implications of insurance status further.

In the literature, studies utilizing a spectrum of methods ranging from observational to cohort to quasi-experimental have suggested that health insurance has a positive effect on health outcomes [2]. A 1993 cohort study found that those without insurance were 1.25 times more likely to die after adjusting for other factors [3]. A 2009 cohort study found that those without insurance were 1.4 times more likely to die [4]. This finding was confirmed by a 2017 review of 18 internal medicine studies [5]. In fact, insurance status has a stronger association with mortality than other significant factors such as race [6].

However, the literature is limited on the relationship between insurance status and hospital outcomes in patients hospitalized with disease and disorders of the nervous system. A 2015 study found that insurance status is correlated with worse self-reported mental health in neurological patients [7]. This study aims to find an association between the lack of insurance and hospital outcomes among patients with diseases and disorders of the nervous system in the United States and investigate the factors associated with lack of insurance.

## Materials and methods

This retrospective cross-sectional study utilized data from the National Inpatient Sample (NIS) database, a component of the Healthcare Cost and Utilization Project (HCUP). NIS contains the inpatient stays information annually and reflects discharges (i.e., the hospital stay) from the US community hospitals including short-term, non-Federal, general and other specialty hospitals excluding hospital units of other institutions, long-term care facilities such as rehabilitation, psychiatric, and alcoholism, and chemical dependency hospitals. The NIS approximates a 20% stratified sample of the hospitals represented in the HCUP database. These stratified data are weighted to generate nationally representative estimates. Individual records in the HCUP databases represent discrete hospital discharges and not necessarily discrete patients [8]. Using the NIS database, the researchers identified patients hospitalized for nervous system diseases and disorders from 2005 to 2014 in the United States by using Major Diagnostic Category (MDC) [9]. Due to the change of ICD-9 to ICD-10 in the middle of 2015 and lack of continuity in some of the outcomes, we decided no to include the year 2015 and after.

The MDCs as described by the Centers for Medicare and Medicaid Services (CMS) are formed by dividing all possible principal diagnoses (from ICD-9-CM) into 25 organ system diagnosis areas. The principal Disease and Disorders of the Nervous System (MDC-01), includes MS-DRGs from 020 to 103. The list of these MS-DRGs is described elsewhere by CMS (see Appendix) [9]. The University of Texas Health at San Antonio exempted this analysis from full review by the Institutional Review Board.

To provide a robust sample across multiple categories, the data were combined across years. Insurance status, type of insurance, hospital mortality rate, and hospital length of stay (LOS) were compared across sex, age, race, geographic region, type of hospital, zip code quartile representing socioeconomic status, whether the hospital visit occurred on the weekend, and the following physician-evaluation variables: the presence of any comorbidity, loss of function, and the likelihood of dying.

Unadjusted and adjusted associations of independent variables and risk factors of mortality were measured using univariate and multivariable (multiple logistic regression) analysis. Variable of interest was the type of insurances which was created based on patients’ first insurance payer into two main groups of insured and uninsured. The HCUP categories Medicare, Medicaid, private (including HMO and other insurances), considered as the insured group. On the other hand self-pay or no charge considered uninsured based on HCUP definitions. A full model includes variables, sex, age, race, being admitted on a weekend, hospital region, comorbidities, being admitted to a teaching hospital, likelihood of dying, loss of function, and zip code quartile that could represent patients’ socioeconomic status. The types of insurances, outcomes, and variables of interest are described in detail in the handbook of HCUP [8].

Odds ratios (ORs) and 95% confidence intervals (CIs) were calculated using the multivariable logistic regression. All P-values were two-sided, and statistical analysis was conducted assuming a two-sided 5% level of significance.

We compared the hospital mortality rate of uninsured and insured patients and the hospital mortality rate across different insurance types. The results were overlaid on other significant correlates of mortality rate to illustrate that age, presence of comorbidity, loss of function, and the likelihood of dying were the most significant correlates with hospital mortality rate aside from insurance status. A multivariate analysis was run to adjust for the effects of age, presence of comorbidity, loss of function, and the likelihood of dying, and t-tests were performed on the adjusted dataset. Statistical software SPSS version 19 was used (IBM SPSS Statistics for Windows, Armonk, NY: IBM Corp).

## Results

We analyzed 10 years (2005-2014) of data consisting of 4,737,999 total hospital discharges with a primary diagnosis of diseases and disorders of the nervous system in the NIS for all payers combined in the United States, the annual mean of 443,146 discharges. Among these discharges, 2,423,031 (51.3%) were covered by Medicare 1,277,150 (27.1%) by private insurance, 597,656 (12.7%) by Medicaid, and 266,321 (5.69%) were uninsured. Approximately, 3.21% of the cohort, included either other minor insurances or missing data.

The study cohort had 40.61% uninsured and 53.03% insured female (prevalence ratio (PR) 0.77, P<0.001), and 59.39% uninsured and 46.97% insured male (PR 1.26, P<0.001).

The mean age of uninsured and insured patients was 44.5±16.1 and 60.1±23.2 years old (P<0.001), respectively. The prevalence ratio between uninsured vs. insured patients regarding age ranged from 0.11 in patients aged ≥65 to 2.80 in 18-45 years old. In individuals under age 18, the prevalence of being uninsured was less than being insured (PR 0.51, P<0.001).

Considering ethnicity, the highest prevalence ratio of uninsured vs. insured was in Hispanics (PR 1.96, P<0.001), then in blacks (PR 1.6, P<0.001), and the lowest ratio was in whites (PR 0.73, P<0.001) (Table 1).

**Table 1:** Demographics and comparison of selected characteristics by insurance types among patients with nervous system diseases or disorders diagnosis in the United States during 2005-2014. *All insured: (MDR: Medicare; MDD: Medicaid; PRV: private). Uninsured: (SLF: self-pay; NCH: no charge). **Excluded all missing values in some variables. ***Based on quartile classification of the estimated median household income of residents in the patient’s ZIP code. ^†^Prevalence of all uninsured patients vs. all insured patients in each variable's level. ^‡^Comparison between all insured and uninsured in each variable.

Variables**		Insurances* (%)			
		All insured	Uninsured	Prevalence ratio^†^	P-value^‡^
Sex	Female (n=2,469,443)	53.03	40.61	0.77	<0.001
	Male (n=2,249,772)	46.97	59.39	1.26	
Age	<18 (n=336,365)	7.32	3.74	0.51	<0.001
	18-45 (n=810,204)	15.57	43.54	2.80	
	46-65 (n=1,304,999)	26.46	47.09	1.78	
	≥65 (n=2,272,596)	50.64	5.63	0.11	
Race	White (n=2,813,680)	70.79	51.63	0.73	<0.001
	Black (n=607,398)	14.55	23.21	1.60	
	Hispanic (n=379,954)	8.92	17.48	1.96	
	Asian (n=89,493)	2.22	2.17	0.98	
	Native (n=23,966)	0.59	0.70	1.19	
	Other (n=123,173)	2.94	4.81	1.63	
Median household income***	0-25^th^ (n=1,325,211)	28.13	38.51	1.37	<0.001
	26-50^th^ (n=1,187,934)	25.63	27.46	1.07	
	51-75^th^ (n=1,108,999)	24.19	21.12	0.87	
	76-100^th^ (n= 995,028)	22.06	12.92	0.59	
Disposition	Routine (n=2,718,161)	56.35	77.06	1.37	<0.001
	Short-term hospital (n=121,632)	2.59	2.29	0.88	
	Other transfers (n=1,172,602)	25.80	8.35	0.32	
	Home health care (n=481,539)	10.55	4.21	0.40	
	Against medical advice (n=53,728)	0.96	4.05	4.20	
	Died in hospital (n=174,513)	3.67	4.00	1.09	
	Discharged alive1.09 (n=3,771)	0.08	0.04	0.53	
Severity of illness (loss of function)	Minor loss of function (n=2,220,629)	45.78	66.78	1.46	<0.001
	Moderate loss of function (n=1,536,188)	33.36	17.80	0.53	
	Major loss of function (n=635,289)	13.77	7.84	0.57	
	Extreme loss of function (n=335,475)	7.07	7.55	1.07	
Risk of mortality (likelihood of dying)	Minor likelihood of dying (n=1,201,035)	24.88	34.13	1.37	<0.001
	Moderate likelihood of dying (n=1,973,626)	41.79	40.85	0.98	
	Major likelihood of dying (n=1,226,328)	26.38	18.53	0.70	
	Extreme likelihood of dying (n=326,592)	6.93	6.45	0.93	
Died in hospital	Yes (n=174,513)	3.67	4.01	1.09	<0.001
	No (n=4,551,433)	96.33	95.99	1.00	

The prevalence of uninsured patients was significantly correlated to socioeconomic status (SES) measured by zip code quartile. The lower the SES, the lower the prevalence of insured patients (0-25th quartile, PR 1.37, P<0.001; 26-50th quartile, PR 1.07, P<0.001; 51-75th quartile, PR 0.87, P<0.001; 76-100th quartile, PR 0.59, P<0.001) (Table 1).

Data on discharge disposition revealed that 4% of patients without insurance left the hospital against medical advice, which was significantly higher than insured individuals (PR 4.2, P<0.001) (Table 1).

Length of stay (LOS) and hospital charges

The mean LOS for patients with nervous system diseases was significantly shorter for uninsured than for insured patients (4.79 days vs. 4.96 days, P<0.001). The longest staying was related to Medicaid beneficiaries (5.88±12.53 days) and the shortest LOS was within private insurances (4.45 days±6.61).

The mean charges per hospital did not differ significantly between uninsured and insured patients ($40,742±70,651 vs. $40,782±72,625, P=0.776).

Hospital mortality rate

**Figure 1:**
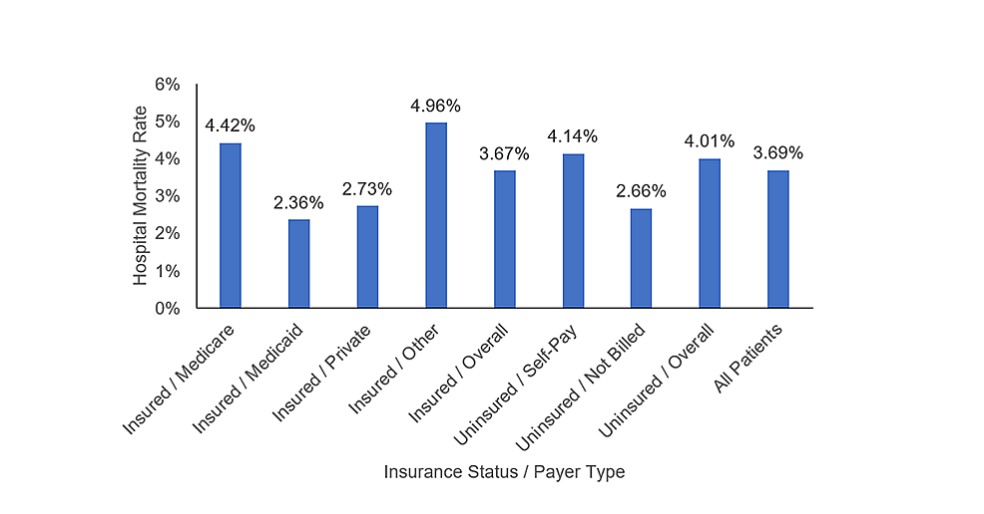
Hospital mortality rate by insurance status and payer type in the United States, 2005-2014.

During the study period, the overall mortality of the cohort was 3.69% with a higher hospital mortality rate among uninsured patients (Figure 1). And this difference is statistically significant (PR 1.09 [4.01% vs. 3.67%], P<0.001).

In the multivariate analysis, the older the age group was, the more difference was observed in mortality rate between uninsured and insured patients (age more than 65 years old, aOR 4.74, [95%CI: 4.52-4.97], P<0.001) (Table 2).

**Table 2:** Hospital mortality rate by selected characteristics and insurance types among patients with nervous system diseases or disorders diagnosis in the United States during 2005-2014. *MDR: Medicare; MDD: Medicaid; PRV: private; SLF: self-pay; NCH: no charge. **Excluded all missing values in some variables. ***Based on quartile classification of the estimated median household income of residents in the patient’s ZIP code. ^†^Odds of mortality of all uninsured patients vs. all insured patients in each variable's level.

				Univariate			Multivariate		
Variables**		All insured*	Uninsured*	Odds ratio^†^		P-value	Odds ratio^†^		P-value
					95%CI			95%CI	
Sex	Female (n=2,469,443)	3.54	3.66	1.03	1.00-1.06	0.052	1.05	1.03-1.06	<0.001
	Male (n=2,249,772)	3.84	4.25	1.11	1.08-1.14	<0.001	Ref		
Age	<18 (n=336,365)	0.82	1.66	2.02	1.72-2.37	<0.001	Ref		
	18-45 (n=810,204)	1.56	2.68	1.72	1.65-1.79	<0.001	2.04	1.94-2.14	<0.001
	46-65 (n=1,304,999)	2.86	4.52	1.58	1.54-1.63	<0.001	3.56	3.40-3.74	<0.001
	≥65 (n=2,272,596)	5.17	11.42	2.21	2.10-2.32	<0.001	4.74	4.52-4.97	<0.001
Race	White (n=2,813,680)	3.79	4.10	1.08	1.05-1.11	<0.001	Ref		
	Black (n=607,398)	2.99	3.53	1.18	1.12-1.24	<0.001	0.75	0.74-0.77	<0.001
	Hispanic (n=379,954)	3.11	3.60	1.16	1.10-1.23	<0.001	0.94	0.92-0.96	<0.001
	Asian (n=89,493)	5.39	6.11	1.13	1.00-1.27	0.029	1.16	1.12-1.20	<0.001
	Native (n=23,966)	3.36	2.95	0.88	0.65-1.18	0.375	0.89	0.82-0.97	0.012
	Other (n=123,173)	3.80	4.58	1.21	1.10-1.33	<0.001	1.02	0.99-1.06	0.109
Weekend	Yes (n=1,041,554)	4.41	4.42	1.00	0.98-1.02	0.845	1.07	1.06-1.09	<0.001
	No (n=3,686,892)	3.47	3.85	1.11	1.07-1.15	<0.001	Ref		
Hospital region	Northeast (n=915,356)	3.65	3.51	0.96	0.91-1.02	<0.001	1.04	1.02-1.06	<0.001
	Midwest (n=1,065,588)	3.36	3.63	1.08	1.03-1.33	<0.001	0.88	0.87-0.90	<0.001
	South (n=1,861,093)	3.71	4.02	1.08	1.05-1.11	<0.001	0.99	0.98-1.01	0.737
	West (n=886,755)	3.99	4.87	1.22	1.16-1.28	<0.001	Ref		
Having any comorbidity	Yes (n=3,871,191)	3.98	4.32	1.09	1.07-1.11	<0.001	2.23	2.18-2.27	<0.001
	No (n=857,601)	2.24	3.11	1.39	1.33-1.45	<0.001	Ref		
Hospital teaching	Urban teaching (n=427,209)	3.82	2.76	0.72	0.70-0.74	<0.001	Ref		
	Urban non-teaching (n=1,645,315)	3.43	3.34	0.97	0.93-1.01	0.153	1.02	1.007-1.03	0.002
	Rural (n=2,631,259)	3.79	4.50	1.19	1.09-1.30	<0.001	1.44	1.41-1.48	<0.001
Severity of illness (loss of function)	No class specified (n=1,211)	1.46	18.29	12.53	5.73-27.42	<0.001	-		
	Minor loss of function (n= 2,220,629)	0.25	0.19	0.76	0.68-0.85	<0.001	0.006	0.0057-0.039	<0.001
	Moderate loss of function (n=1,536,188)	1.68	1.71	1.02	0.95-1.09	0.611	0.038	0.038-0.039	<0.001
	Major loss of function (n=635,289)	5.63	7.14	1.27	1.20-1.34	<0.001	0.13	0.12-0.13	<0.001
	Extreme loss of function (n=335,475)	31.44	39.98	1.27	1.23-1.31	<0.001	Ref		
Risk of mortality (likelihood of dying)	No class specified (n=1,211)	1.46	18.29	12.53	5.73-27.42	<0.001	1		
	Minor likelihood of dying (n=1,201,035)	0.59	0.37	0.63	0.56-0.70	<0.001	0.74	0.72-0.77	<0.001
	Moderate likelihood of dying (n=1,973,626)	1.10	0.91	0.83	0.78-0.88	<0.001	0.90	0.87-0.93	<0.001
	Major likelihood of dying (n=1,226,328)	5.27	7.35	1.39	1.34-1.44	<0.001	1.11	1.07-1.15	<0.001
	Extreme likelihood of dying (n=326,592)	24.15	33.18	1.37	1.33-1.42	<0.001	Ref		
Median household income***	0-25^th^ (n=1,325,211)	3.71	4.07	1.10	1.06-1.14	<0.001	1.03	1.01-1.05	<0.001
	26-50^th^ (n=1,187,934)	3.70	3.87	1.05	1.01-1.09	0.026	1.02	1.001-1.04	0.005
	51-75^th^ (n=1,108,999)	3.56	3.76	1.06	1.01-1.11	0.012	0.97	0.95-0.99	0.006
	76-100^th^ (n=995,028)	3.69	4.34	1.18	1.12-1.25	<0.001	Ref		

Considering ethnicity, in multivariate analysis, Asians had higher mortality rate in uninsured patients (aOR:1.16, [95%CI: 1.12-1.20], P<0.001), while in other ethnicities uninsured patients had lower mortality (Black: aOR 0.75, [95%CI: 0.74 - 0.77], P<0.001; Hispanic: aOR 0.94, [95%CI: 0.92-0.96], P<0.001; Native American: aOR 0.89, [95%CI: 0.82-0.97], P<0.001) (Table 2).

Uninsured patients had a higher mortality rate than insured ones in both patients with comorbidity and without comorbidity and this difference was more in those with comorbidity (aOR 2.23, [95%CI: 2.18-2.27], P< 0.001) (Table 2).

Patients with extreme loss of function (OR 1.27, [95%CI: 1.23-1.31], P< 0.001) had a larger difference in all-cause mortality rate than patients with minor loss of function (OR 0.76, [95%CI: 0.68-0.85], P< 0.001). Those with major or extreme likelihood of dying (OR 1.37, [95%CI: 1.33-1.42], P< 0.001) had a larger difference in all-cause mortality rate than those with minor likelihood of dying (OR 0.63, [95%CI: 0.56-0.70], P< 0.001) (Table 2). 

## Discussion

The retrospective cross-sectional study of data consisting of 4,737,999 total hospital discharges was done to compare hospital outcomes in overall 5.69% uninsured patients as compared to the insured patients admitted with the primary diagnosis of disease and disorders of nervous system.

Although the lack of insurance is observed more in populations that are less vulnerable and have less likelihood of dying at the time of admission, the rate of in-hospital mortality is higher in patients without insurance. This finding is in accord with previous studies [4,5,10,11]. However, there is a lack of evidence for the mortality rate in the population with nervous system diseases without health insurance. Our study uniquely suggests that the hospital mortality rate among neurological patients is increased for uninsured patients.

A national study shows that the in-hospital mortality rate was higher in stroke patients without insurance than insured patients [12]. Shen et al. found that mortality risk in uninsured patients with intracerebellar hemorrhage and acute stroke is higher 24% and 56%, respectively, than privately insured patients [13]. We believe the lack of health insurance would increase the fear of the cost of care; therefore, uninsured patients delay seeking medical care, and they may be sicker at the time of admission [3]. Also, financials may cloud judgment to choose certain expensive diagnostic and therapeutic measures.

In the current study, based on multivariate analysis, this discrepancy is more prominent in the following groups: the elderly 65+, Asians, patients with comorbidity, hospitals located in rural areas and Northeast regions, and 0-25th median household income.

In terms of LOS in the hospital due to nervous system diseases and disorders, insured patients have a longer LOS as compared to uninsured patients in our study. It is consistent with previous studies that showed LOS is shorter in uninsured patients [14-16]. In our study, Medicaid beneficiaries have the longest stay among insured patients which is 5.88±12.53 days. In another study, the mean of LOS was 5.87 days in Medicaid patients, which was longer than uninsured patients and patients insured by Blue Cross Blue Shield [16]. The shorter LOS in uninsured patients could be multifactorial. For example, the incidence of leaving the hospital against medical advice is more common in the uninsured and this early leaving can lead to a shorter LOS in this population. This reason is also backed up by our finding of a higher rate of disposition against medical advice in the uninsured. Another potential explanation for shorter LOS in the uninsured population could be that hospitals try to discharge the non-funded patients during a shorter period to lower their expenses.

Our study showed that although Hispanics and blacks are the most uninsured population, however, Asians have the highest mortality in the cohort. The disparity in insurance status is noticeable in different ethnicities, and the gap is especially high in the Hispanic and African American populations, which is consistent with the literature. In other words, racial minorities are less likely to be covered by health insurance [6,17,18] This can be due to the higher unemployment rate and lower access to private insurance through employment and marriage in African Americans and Hispanics [18]. However, the less mortality in Hispanics and blacks compared to the whites is not well understood. It can be due to genetic differences or seeking medical attention regardless of lack of insurances.

In the current study, 59.39% of uninsured patients were male, and the absolute mortality percentage was higher in males in both insured and uninsured, although the adjusted mortality was slightly higher in females. This is consistent with a study done in 2018 that discovered the uninsured rate in men and women in the US was 14% and 11%, respectively [19]. Although women are less likely to have employment-based coverage, there is a greater likelihood of becoming qualified for Medicaid due to pregnancy or as parents of young children [20]. The adjusted higher mortality rate in females also has been discussed in the literature especially due to their underlying medical conditions, genetics, and a higher mortality rate in general [21-23].

Our results showed that there is an association between the lower-income patients and a higher rate of lack of insurance and moreover patients are more likely to die if they are uninsured. Low household income proportionately affects insurance status, which strongly affects patients to self-discharge against medical advice, which puts them in the most vulnerable conditions. In several studies, it has been shown that insurance status is one of the predictors of self-discharge, which is highest among the uninsured, and amongst the insured is more in Medicaid beneficiaries. Unfortunately, the lower-income population have more difficulty in affording buying insurances and this is not limited only to the neurological patient population and this topic has been a center of attention and debate for the governments and a hot topic usually for each political party in the United States [24-26].

Our study showed although most of the elderly are insured, uninsured elderly had higher adjusted mortality. The higher mortality of uninsured elderly could be multifactorial. First, the elderly in general have a higher rate of death due to more medical comorbidities. Second, the uninsured elderly do not have enough access to healthcare and medications and by the time that they admit for the nervous system disease at the hospitals, they are more likely chronically ill and suffer from uncontrolled medical conditions such as high blood pressure and diabetes, and die with a higher likelihood [13,27]. Children under the age of 18 and the elderly over the age of 65 have the lowest rate of being uninsured, which can be due to social programs such as CHIP and Medicare for the above categories. Overall, the uninsured patients having nervous system diseases are younger (44.49 years of age) vs. the insured (60.08 years of age); however, surprisingly there is no statistical difference between insured and uninsured populations in terms of hospital charges. The equivalent billable charges suggest that the number of procedures and amount of care provided were likely similar for uninsured and insured patients. Accordingly, based on our study, the discrepancy in LOS is not necessarily because of expenses but maybe due to age.

## Conclusions

Overall, the findings suggest that 5.69% of disease and disorders of nervous system discharges are uninsured and lack of health insurance is correlated with worse hospital outcomes in this population in the United States. This may be due to cumulative effects of delaying healthcare services and low socioeconomic condition which has an impact on the severity of outcomes in the nervous system disorders. Lack of health insurance appears to have the greatest negative impact on the elderly, racial minorities, and those rated as having the highest loss of function and likelihood of mortality. A follow-up cohort study could investigate the directionality of a possible causal relationship.

## Appendices

**Table 3:** Appendix contains a list of each All Patient-Refined Diagnosis-Related Groups (APR-DRG) with a specification of the MDC-01 and whether the APR-DRG is medical or surgical. The letter “M” is used to designate a medical APR-DRG and the letter “P” is used to designate a surgical APR-DRG.

DRG	MED-SURG	MDC	Long description
020	P	01	CRANIOTOMY FOR TRAUMA
021	P	01	CRANIOTOMY EXCEPT FOR TRAUMA
022	P	01	VENTRICULAR SHUNT PROCEDURES
023	P	01	SPINAL PROCEDURES
024	P	01	EXTRACRANIAL VASCULAR PROCEDURES
026	P	01	OTHER NERVOUS SYSTEM & RELATED PROCEDURES
040	M	01	SPINAL DISORDERS & INJURIES
041	M	01	NERVOUS SYSTEM MALIGNANCY
042	M	01	DEGENERATIVE NERVOUS SYSTEM DISORDERS EXC MULT SCLEROSIS
043	M	01	MULTIPLE SCLEROSIS & OTHER DEMYELINATING DISEASES
044	M	01	INTRACRANIAL HEMORRHAGE
045	M	01	CVA & PRECEREBRAL OCCLUSION W INFARCT
046	M	01	NONSPECIFIC CVA & PRECEREBRAL OCCLUSION W/O INFARCT
047	M	01	TRANSIENT ISCHEMIA
048	M	01	PERIPHERAL, CRANIAL & AUTONOMIC NERVE DISORDERS
049	M	01	BACTERIAL & TUBERCULOUS INFECTIONS OF NERVOUS SYSTEM
050	M	01	NON-BACTERIAL INFECTIONS OF NERVOUS SYSTEM EXCEPT VIRAL MENINGITIS
051	M	01	VIRAL MENINGITIS
052	M	01	NONTRAUMATIC STUPOR & COMA
053	M	01	SEIZURE
054	M	01	MIGRAINE & OTHER HEADACHES
055	M	01	HEAD TRAUMA WITH COMA >1 HR OR HEMORRHAGE
056	M	01	BRAIN CONTUSION/LACERATION & COMPLICATED SKULL FX, COMA < 1 HR OR NO COMA
057	M	01	CONCUSSION, CLOSED SKULL FX NOS, UNCOMPLICATED INTRACRANIAL
